# A five-antigen Esx-5a fusion delivered as a prime-boost regimen protects against *M.tb* challenge

**DOI:** 10.3389/fimmu.2023.1263457

**Published:** 2023-10-05

**Authors:** Elena Stylianou, Nawamin Pinpathomrat, Oliver Sampson, Alexandre Richard, Marcellus Korompis, Helen McShane

**Affiliations:** The Jenner Institute, University of Oxford, Oxford, United Kingdom

**Keywords:** tuberculosis, vaccines, mucosa, protection, viral-vector, BCG, subunit, Esx

## Abstract

The development of tuberculosis (TB) vaccines has been hindered by the complex nature of *Mycobacterium tuberculosis* (*M.tb*) and the absence of clearly defined immune markers of protection. While Bacillus Calmette-Guerin (BCG) is currently the only licensed TB vaccine, its effectiveness diminishes in adulthood. In our previous research, we identified that boosting BCG with an intranasally administered chimpanzee adenovirus expressing the PPE15 antigen of *M.tb* (ChAdOx1.PPE15) improved its protection. To enhance the vaccine’s efficacy, we combined PPE15 with the other three members of the Esx-5a secretion system and Ag85A into a multi-antigen construct (5Ag). Leveraging the mucosal administration safety of ChAdOx1, we targeted the site of *M.tb* infection to induce localized mucosal responses, while employing modified vaccinia virus (MVA) to boost systemic immune responses. The combination of these antigens resulted in enhanced BCG protection in both the lungs and spleens of vaccinated mice. These findings provide support for advancing ChAdOx1.5Ag and MVA.5Ag to the next stages of vaccine development.

## 1 Introduction

Tuberculosis (TB) remains one of the leading causes of death worldwide (1). TB is caused by *Mycobacterium tuberculosis* (*M.tb*), which is transmitted via aerosol droplets when an infected person coughs or sneezes, primarily causing disease in the lungs (2). Although *M.tb* has existed for thousands of years, our understanding of its pathogenicity is incomplete (3, 4). Bacillus Calmette-Guerin (BCG) is the only licensed vaccine against TB, and although it reliably protects against childhood forms of TB, its efficacy is inconsistent and wanes in adults, who are responsible for 90% of cases (1, 5).

Subunit vaccines, designed to boost neonatal BCG vaccination, exploit the childhood efficacy of BCG, as well as its ability to induce non-specific protection against other pathogens (6). There are six subunit vaccines currently being evaluated in clinical trials, either protein–adjuvant combinations or recombinant viral vectors (7). The ability of a subunit vaccine to confer protection against TB disease was recently demonstrated with the M72/AS01_E_ candidate vaccine, where a fusion of two mycobacterial antigens delivered with the AS01_E_ adjuvant provided 49.7% efficacy against progression to active disease in latently infected *M.tb* adults (8). The selection of antigens to include in subunit vaccine candidates is a critical step in the process of developing a subunit vaccine, as well as the combination of antigens to ensure that there is a synergistic effect. Eleven mycobacterial antigens have been tested as protein adjuvants in clinical trials to date, but only two, Ag85A and ESAT-6, have been tested in recombinant viral vectors (9–13).

We previously evaluated the protective efficacy of several mycobacterial antigens, shown to be well-recognized by TB patients, those with latent *M.tb* infection and *M.tb*-infected mice (14, 15). We utilized the natural tropism for the lung of a chimpanzee adenovirus (ChAdOx1), as a delivery vector for these antigens, to induce specific immune responses at the primary site of *M.tb* infection. One antigen, PPE15 (Rv1039c), provided protection in both the lung and spleen of vaccinated mice, both when administered alone and as a boost to BCG (14). PPE15 belongs to the Esx-5a family of proteins of *M.tb. M.tb* has five type VII secretion systems (T7SS) also known as Esx systems. These are specialized secretion systems used by the bacterium to secrete virulence factors such as EsxA (ESAT-6), considered one of the main reasons for the success of *M.tb* as a pathogen (16, 17). Esx-5 is believed to be the more recently duplicated, present only in slow-growing mycobacteria (including most pathogenic) and responsible for the secretion of many PE/PPE proteins as well as the acquisition of nutrients (18–20). The Esx-5 system is composed of structural core proteins, esx conserved components (ecc), a conserved subunit with protease activity (MycP), two secreted Esx proteins, and genes encoding PE/PPE proteins (21). Recent studies have focused on the architecture of Esx-5 that could help understand its function and help develop novel drugs and vaccines (22–24). A region of four genes (*esx-gene cluster*), two encoding ESAT-6 like proteins, esx, and two PE/PPE proteins was duplicated three times from Esx-5, at random locations in the *M.tb* genome (25). These regions are referred to as Esx-5a-c (21). Esx-5a spans from loci Rv1037c-Rv1040c and contains genes, *esxI, esxJ, ppe15*, and *pe8.* EsxI and EsxJ are both secreted proteins, as they were found in the culture filtrates of *M.tb* (26). Esx-5a has been shown to support the Esx-5 system in the secretion of some *M.tb* proteins, and deletion of this region resulted in reduced inflammasome activation, which might be attributed to the lack of secreted proteins (21, 27). The function of the Esx-5b and Esx-5c regions remains unknown.

In this study, we combined the protective PPE15 antigen with the other members of the Esx-5a family to investigate whether there was a synergistic protective effect. These antigens, along with the well-characterized Ag85A of *M.tb*, were evaluated alone or as a fusion protein, to protect against *M.tb* infection, when expressed by ChAdOx1, both alone and when boosted by a recombinant modified vaccinia Ankara virus (MVA) expressing the same antigens. We found that two antigens improved the protective efficacy of BCG, and the combination of five antigens (PPE15, PE8, EsxI, EsxJ, and Ag85A) resulted in a synergistic protective effect. In conclusion, ChAdOx1.5Ag and MVA.5Ag improved the protective efficacy of BCG and merit further evaluation in the more stringent non-human primate model.

## 2 Materials and methods

### 2.1 Viral vector generation

Attenuated Chimpanzee Adenovirus Oxford (ChAdOx1) was modified to express each antigen of the Esx-5a secretion system separately. The fusion of four antigens, PE8, PPE15, EsxJ, and EsxI, was prepared using GeneArt Technology (ThermoFisher Scientific, UK). Ag85A was subsequently added at the C-terminus by PCR. The generation of ChAdOx1 and MVA constructs was performed as previously described (14, 28). Briefly, the antigens were cloned into a Gateway entry vector, under a modified human cytomegalovirus major immediate early promoter (CMV). This was followed by recombination into the ChAdOx1 destination DNA BAC vector, with E1 and E3 regions deleted and E4 modified. The ChAdOx1 genome was excised with PmeI, and the virus was rescued and propagated in human embryonic 293 (HEK293 cells). For MVA, antigens were subcloned into a poxvirus shuttle vector under the control of the vaccinia virus p7.5 promoter, which was later recombined at the TK locus of MVA in chicken embryo fibroblasts (CEFs). Rescue and purification of the viruses were performed by the Viral Vector Core Facility, University of Oxford.

### 2.2 Ethics statement

All procedures were performed in accordance with the UK Animals (Scientific Procedures) Act 1986, under project license number 30/2889 and P9804B4F1, granted by the UK Home Office.

### 2.3 Mice and Immunizations

Six- to 8-week-old female CB6F1 mice were purchased from Charles Rivers, UK.

Mice were vaccinated with BCG-Pasteur at 3.5 × 10^5^ colony-forming units (CFU) intradermally (i.d.) administered in a final volume of 50 μL. BCG was prepared in-house, in 7H9 broth (Becton Dickinson, UK) supplemented with 10% middlebrook albumin dextrose catalase and 0.05% polysorbate 80 (Becton Dickinson, UK).

Vaccinations with ChAdOx1 constructs were performed either i.d. or intranasally (i.n.), with 1 × 10^8^ infectious units (ifu) in a 50-μL volume, diluted in endotoxin-free PBS (Merck Life Science, UK). For the “combo” group, 1 × 10^8^ ifu of each ChAdOx1 construct were mixed into a single administration. MVA constructs were administered i.d. at 5 × 10^6^ plaque-forming units (pfu) per dose in a final volume of 50 μL. For BCG-boosting experiments, 10 or 14 weeks were allowed between BCG-prime and booster vaccines.

### 2.4 Immunogenicity

Bronchoalveolar lavage (BAL), lung, and spleen cells were extracted from naïve and vaccinated mice. Lungs were perfused with PBS, chopped into small pieces, and digested with DNase/Collagenase (Merck Life Science, UK).

ELISpot: Multiscreen-IP ELISpot plates (Millipore, Meck Life Science) were coated with anti-mouse IFNγ and developed using the ELISpot Flex kit (Mabtech, USA). Cells were stimulated with media alone or with a final concentration of 2 μg/mL of each peptide pool (PE8, PPE15, esxJ, esxI, and Ag85A) of 15-mer peptides overlapping by 11 amino acids and spanning the whole antigen sequence (Peptide Protein Research, UK) or 10 μg/mL of protein purified derivative (PPD-T) (AJ vaccines). Plates were read using an ELISpot reader (AID ELISpot) and presented as spot-forming units (SFU) per million cells, by subtracting the number of spots in the unstimulated (media) from the peptide stimulated wells and correcting for the number of cells in the well.

Flow cytometry: lung and spleen cells were stimulated as above for 2 h, before the addition of Golgi plug (BD Biosciences) for a further 4-h stimulation at 37°C followed by incubation overnight at 4°C. Cells were stained the following day with live/dead fixable stain (ThermoFisher Scientific) for 10 min followed by α-CD45R, α−TCRαβ, α-CD8 (eBioscience), and α-CD4 (BioLegend, Inc.). Cells were permeabilized with Cytofix/Cytoperm and stained intracellularly with α-IFNγ, -TNFα, -IL2, and -IL17 (eBioscience). Samples were run on an LSR II flow cytometer, and the data were analyzed using FlowJo (TreeStar Inc., Ashland, OR, USA).

Enzyme-linked immunosorbent assay (ELISA) was used to measure Ag85A and PPE15-specific IgG responses in the serum. Maxisorp 96-well plates (ThermoFisher Scientific) were coated with 2 μg/mL of Ag85A or PPE15 protein in PBS and incubated overnight. Plates were washed and blocked with 2.5% BSA/PBS, followed by a 2-h incubation with serially diluted serum samples (starting dilution 1:50) prepared in PBS/Tween. Following washing, alkaline phosphatase-conjugated anti-IgG (Sigma-Aldrich) diluted in PBS/Tween was added and incubated for 1 h at RT. Development was with 1 mg/mL of 4-nitrophenylphosphate tablet (Sigma-Aldrich) diluted in diethanolamine buffer (ThermoFisher Scientific). Optical density (OD) was measured at 405 nm (Gen5 software) using a spectrophotometer (BioTeK Microplate Reader). OD was the final readout.

For the immunogenicity of ChAdOx1-expressing Esx5a antigen experiments, we included ChAdOx1.esxI, esxJ, and PE8 groups. For flow cytometry the ChAdOx1.PPE15 group was also included. Specific responses were determined by subtracting antigen-specific responses from the unstimulated (media) wells. To determine the immunogenicity of the 5Ag ChAdOx1 and MVA constructs, animals were vaccinated with ChAdOx1.5Ag or MVA.5Ag. Specific responses were determined as above. For the heterologous prime-boost combinations, groups included C-M, C, and unvaccinated naïve controls. Specific responses were detected by subtracting responses in the stimulated wells compared to unstimulated (media) wells.

### 2.5 Mycobacterial challenge

A Biaera Aero-MP-controlled nebulizer (Biaera Technologies, USA) was used to infect mice with 50–100 CFU of aerosolized *M.tb* Erdman K01 (TMC107, bei resources, USA) at an airflow rate of 12 L/min and a pressure of 20 lb/in^2^ gauge. Infection dose was confirmed 24 h post-infection with two mice. Challenge experiments included a naïve and a BCG vaccinated group as controls.

Lungs and spleen were collected 4 weeks after infection and homogenized in reinforced tubes (Stretton Scientific) and a precellys 24 homogenizer. Homogenates were serially diluted, plated on Modified 7H11 plates (Animal and Plant Health Agency, UK), and incubated at 37°C for 5 weeks.

### 2.6 Statistical analysis

GraphPad Prism (version 8 and 9) was used to create graphs and perform analysis. A Mann–Whitney test was used to compare two groups and Kruskal–Wallis followed by Dunn’s multiple comparison testing for multiple groups.

## 3 Results

### 3.1 Immunogenicity of ChAdOx1-expressing Esx-5a antigens

**Figure 1:**
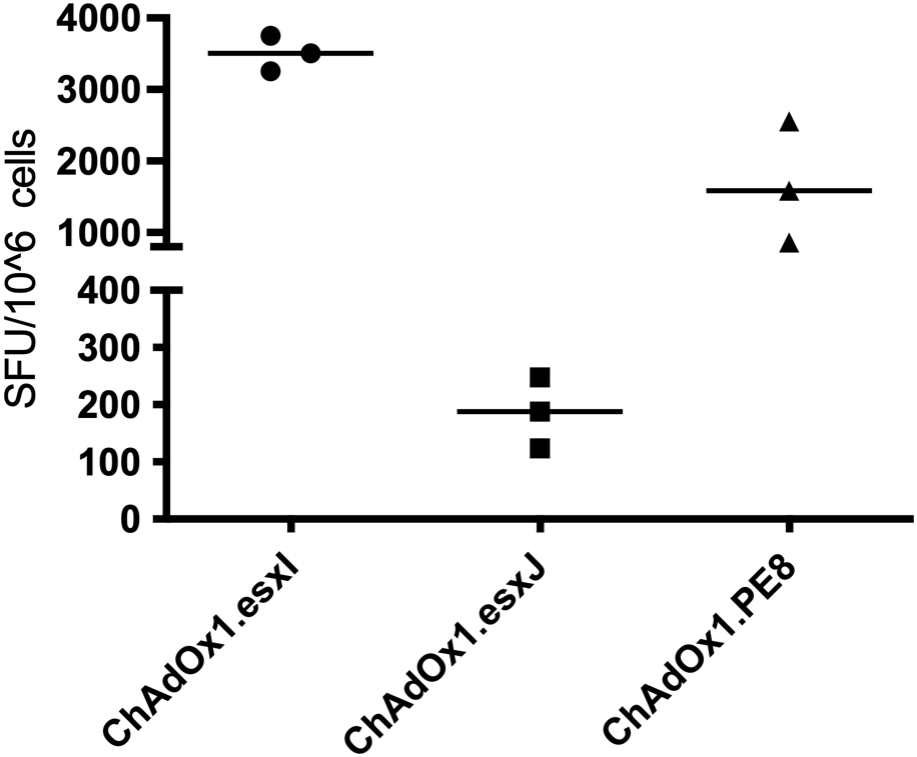
Immune responses following a vaccination with ChAdOx1 vaccines. CB6F1 mice were intradermally vaccinated with ChAdOx1 expressing three antigens of the Esx-5a system. Splenocytes were recovered 2 weeks later and stimulated with peptide pools. Antigen-specific immune responses were measured using IFNγ-ELISpot. Each symbol represents one animal, and the line denotes the median of each group. SFU, spot-forming units.

To confirm antigen expression and test the immunogenicity of the viral vectors, mice were vaccinated with either ChAdOx1.PE8, ChAdOx1.esxI, or ChAdOx1.esxJ, i.d. The immunogenicity of ChAdOx1.PPE15 was previously confirmed (14). The induction of systemic, antigen-specific, interferon-gamma IFNγ responses was detected in *ex vivo* ELISpot assays, 2 weeks following a single vaccination. A strong IFNγ response was induced following ChAdOx1.esxI and ChAdOx1.PE8, and a moderate response after ChAdOx1.esxJ (**Figure 1**).

**Figure 2:**
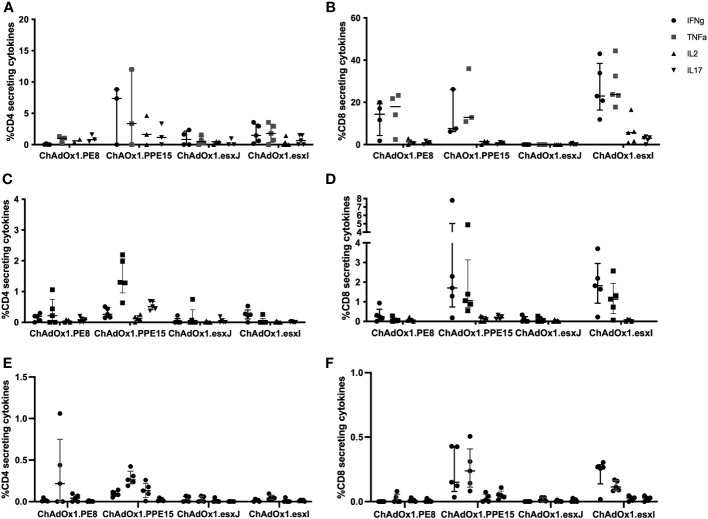
Immunogenicity of mucosally administered ChAdOx1 vaccines. ChAdOx1 expressing the four antigens of the Esx-5a system was administered intranasally to CB6F1 mice. Antigen-specific CD4^+^ Th1 and CD8^+^ immune responses in the **(A, B)** bronchoalveolar lavage (*n* = 3–5), **(C, D)** lung, and **(E, F)** spleen were measured 4 weeks after vaccination. *n* = 5 per group, each symbol represents one animal and the error bars denote the interquartile range.

To assess the capacity to induce a mucosal immune response, viral vectors were administered via the intranasal route (i.n.). ChAdOx1.PPE15 induced strong CD4+ T-cell responses in the BAL, lung, and spleen (**Figures 2A, C, E**). CD4+ T-cell responses were also detected, but in lower percentages with the other three viruses. Strong CD8+ T-cell responses were detected in the BAL, lung, and spleen, following ChAdOx1.PPE15 and ChAdOx1.esxI, and lower responses following ChAdOx1.PE8. There were no detectable responses after ChAdOx.esxJ (**Figures 2B, D, F**). These results indicate that i.n. administration of ChAdOx1 can induce both mucosal and systemic CD4+ and CD8+ responses, and some antigens are more immunogenic than others.

### 3.2 Efficacy of ChAdOx1 Esx-5a vaccine candidates

**Figure 3:**
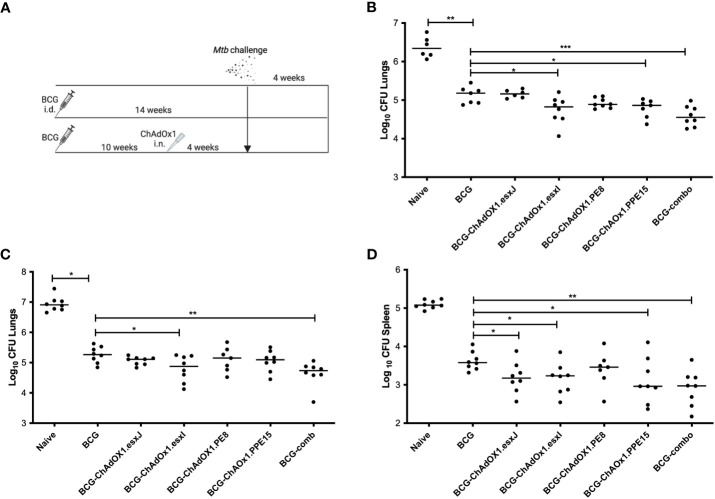
Efficacy of intranasal ChAdOx1 expressing Esx-5a antigens. **(A)** CB6F1 mice were primed with i.d. BCG and boosted with i.n. ChAdOx1 10 weeks later. Unvaccinated (Naïve) and mice vaccinated with BCG were used as controls. Four weeks after the last vaccination, all mice were challenged with aerosol *M.tb*, and 4 weeks later, **(B)** lung was collected for CFU enumeration. **(C)** Lung and **(D)** spleen CFU from repeat challenge. Each dot represents one animal, and the line denotes the median of each group. Kruskal–Wallis followed by multi-comparison testing, *p < 0.05, **p < 0.01, ***p < 0.001. CFU, colony-forming units; i.d., intradermal; i.n., intranasal.

To assess whether the viruses could improve the protective efficacy of BCG, mice were primed with i.d. BCG and boosted with i.n. viruses 10 weeks later (**Figure 3A**). A group of BCG-primed animals received a combination of the four viruses in one administration to determine whether there was a synergistic protective effect (BCG-comb). All mice were challenged with aerosolized *M.tb* and lung and spleen were collected for bacterial enumeration (**Figure 3A**).

BCG-primed animals boosted with i.n. ChAdOx1.PPE15 and ChAdOx1.esxI had a significantly lower lung bacterial count compared to mice vaccinated only with BCG (*p* = 0.03 and *p* = 0.02, respectively) (**Figure 3B**). ChAdOx1.PE8 and ChAdOx1.esxJ did not improve upon BCG-induced protection. The BCG-comb regimen significantly improved BCG efficacy (*p* = 0.0006) and had the lowest median CFU. The experiment was repeated with similar findings (**Figure 3C**). In the spleen, all groups except ChAdOx1.PE8 had a significantly lower bacterial count compared to the BCG group (**Figure 3D**).

### 3.3 Immune responses induced by recombinant ChAdOx1 and MVA vaccine candidates expressing all five antigens as a single fusion

**Figure 4:**
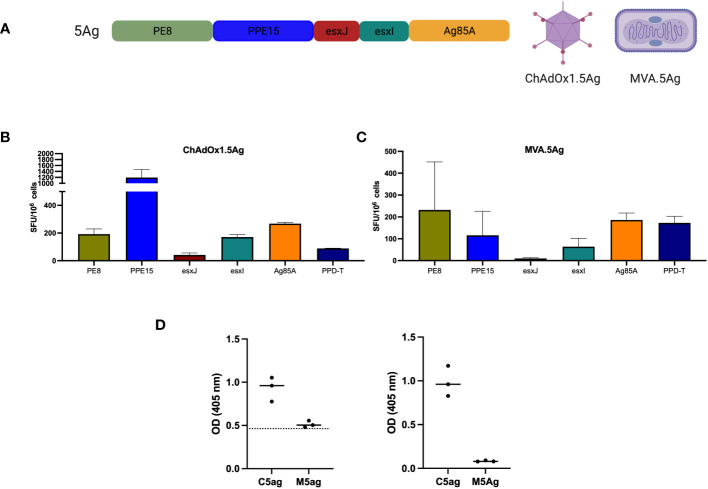
Immunogenicity of ChAdOx1 and MVA expressing a string of five antigens. **(A)** Schematic of the 5-antigen construct. CB6F1 were vaccinated with either **(B)** ChAdOx1.5Ag or **(C)** MVA.5Ag intradermally and were harvested after 2 and 1 week, respectively, for the evaluation of antigen-specific immune responses using IFN-γ ELISpot. Bar represents the median value with an interquartile range of three mice per group. **(D)** PPE15 (left graph) and Ag85A-specific (right) IgG responses were measured using ELISA. Each dot represents one animal (*n* = 3), the line denotes the median value, and the dotted line indicates the background antibody level. SFU, spot-forming units; OD, optical density.

To exclude the possibility that the protective effect of ChAdOx1.comb was due to increased adjuvanticity because of a higher number of viral particles, we developed a ChAdOx1 to express all four antigens of the Esx-5a system, in addition to antigen 85A (Ag85A) as a single fusion protein (ChAdOx1.5Ag) (**Figure 4**). We previously showed that the efficacy of i.n. ChAdOx1.85A could be further improved with i.d. MVA.85A (29, 30). To test whether this was also the case for ChAdOx1.5Ag, we also developed an MVA to express the same five-antigen construct (MVA.5g) (**Figure 4**).

Both i.d. ChAdOx1.5Ag and MVA.5Ag were administered separately and immune responses to each of the five antigens were measured. PPD-T was used as a positive control. IFNγ immune responses were detected for all antigens following splenocyte stimulation, with the lowest immune responses detected for esxJ (**Figures 4B, C**). Antibody-specific responses to PPE15 and Ag85A were measured in the serum of vaccinated animals (**Figure 4D**). ChAdOx1.5Ag-induced antibody responses to both antigens were tested, whereas MVA.5Ag did not induce any detectable antibody response.

### 3.4 Heterologous prime-boost immunization with ChAdOx1 prime-MVA boost

**Figure 5:**
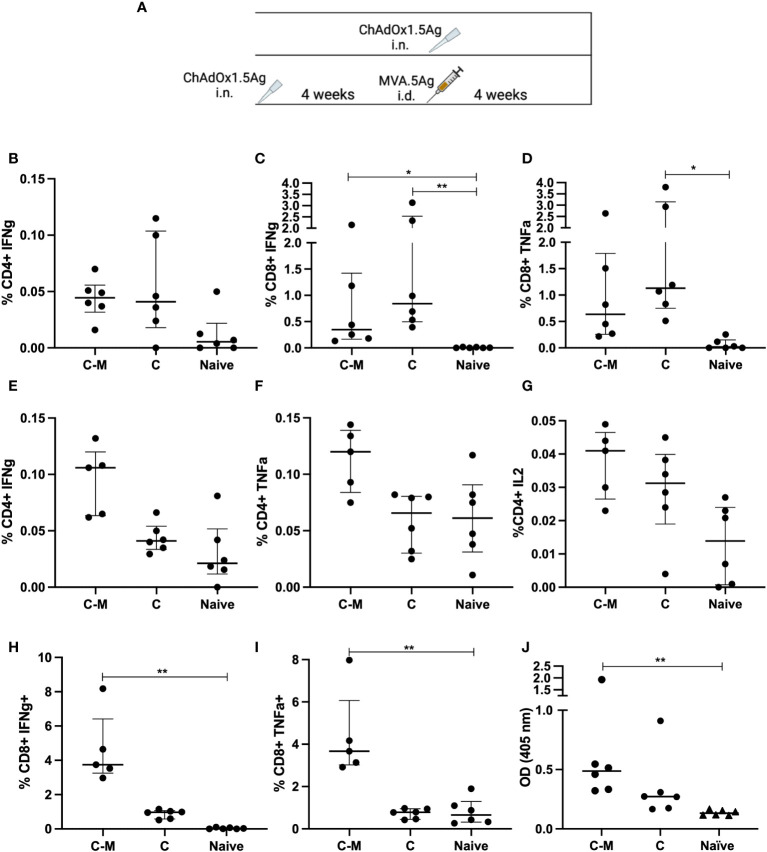
Immune responses following mucosal and prime-boost vaccinations. **(A)** CB6F1 mice were vaccinated intranasally (i.n.) with ChAdOx1.5Ag, with some animals boosted with intradermal MVA.5Ag, and some animals left unvaccinated as per experimental schema. **(B–D)** PPE15-specific CD4^+^ IFNγ **(B)** and CD8^+^ IFNγ and TNFα **(C, D)** were measured in the lung. Vaccine-specific immune responses were also evaluated in the spleen, **(E–G)** CD4^+^ IFNγ, TNFα, IL2, and **(H, I)** CD8^+^ IFNγ and TNFα. **(J)** Ag85A-specific IgG responses were measured by ELISA. *n* = 6 per group. Each dot represents one animal, and the line denotes the median of each group. Kruskal-Wallis followed by multi-comparison testing, *p < 0.05, **p <0.01.

Boosting ChAdOx1 with MVA was previously shown to improve both the immunogenicity and efficacy of either vector alone (29). To explore whether this was the case for the 5Ag constructs, mice were vaccinated i.n. with ChAdOx1.5Ag and boosted with i.d. MVA.5Ag (C-M), 4 weeks later (**Figure 5A**). A group of mice received ChAdOx1.5Ag (C) alone and a group was left unvaccinated. Lung and spleens were collected 4 weeks after the last immunization for the assessment of CD4+ and CD8+ T-cell responses. Stimulation with PPE15 was used as the representative antigen to determine antigen-specific immune responses.

CD4+ IFNγ responses were detected in the lungs of both C-M and C vaccinated animals but there were no detectable CD4+ TNFα and IL2 (**Figure 5B**). Both vaccine regimens induced a comparable high percentage of CD8+ cells, releasing IFNγ and TNFα (**Figures 5C, D**). In the spleen, the percentage of CD4+ T cells releasing IFNγ and TNFα was higher following C-M vaccination, compared to C, although the difference did not reach significance (**Figures 5E, F**). Low but detectable CD4+ IL2 responses were measured in the splenocytes of C-M and C animals (**Figure 5G**). C-M vaccination induced strong CD8+ IFNγ and TNFα responses in the spleen, significantly higher compared to the naïve, but not C vaccinated group (**Figures 5H, I**). IFNγ responses to the remaining antigens were lower compared to PPE15 but followed the same trend (**Supplementary Figure 2**).

Strong Ag85A-specific IgG responses were measured in the serum of C-M vaccinated mice, and detectable but lower responses in mice vaccinated with C (**Figure 5J**). Antibody responses to PPE15 were very weak in both vaccinated groups (data not shown).

The N-terminal part of PPE15 was previously shown to be the most immunogenic and likely protective (14). To determine whether this was still the case following ChAdOx1.5Ag vaccination, cells from the above experiment were stimulated with the two halves of PPE15 (**Supplementary Figure 1**). The N-terminal half induced higher IFNγ responses compared to the second half, in agreement with previous data.

### 3.5 Protective efficacy of ChAOx1.5Ag and MVA.5Ag

**Figure 6:**
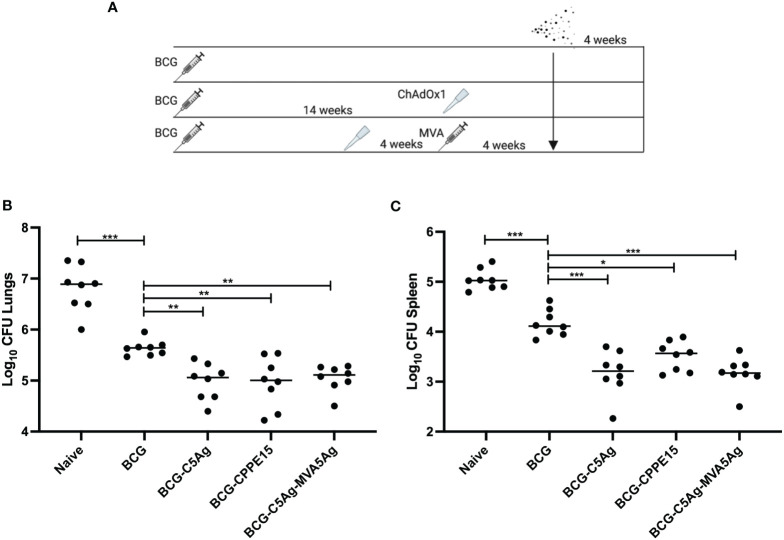
Boosting the protective efficacy of BCG with viral vectors. **(A)** CB6F1 mice were primed with BCG and boosted with intranasal C5Ag or CPPE15, and some animals received a second intradermal boost of MVA.5Ag. All animals were challenged with aerosol *M.tb* and lungs and spleen were harvested at the end of the experiment for bacterial enumeration. **(B)** Lung and **(C)** spleen bacterial load. Each symbol represents one animal, and the line denotes the median value of each group. Statistical differences were calculated using Kruskal–Wallis followed by multi-comparison testing. *p < 0.05, **p < 0.01, ***p < 0.001. CFU, colony-forming units.

As these vaccines are aimed to boost the efficacy of BCG, their ability to improve protection was tested in a BCG prime-boost challenge study. Mice were primed with BCG and boosted with either i.n. C5Ag or CPPE15. Some animals were further boosted with i.d. MVA.5Ag. All mice were challenged with aerosolized *M.tb*, and lung and spleen were collected at the end of the experiment for bacterial enumeration (**Figure 6A**).

In the lung, BCG vaccinated animals had significantly lower bacterial load, compared to unvaccinated mice (**Figure 6B**). All vaccine regimens tested in this study significantly improved the efficacy of BCG, but there were no differences between the groups. Similarly in the spleen, BCG vaccinated animals had a lower bacterial load compared to naïve mice (**Figure 6C**). Boosting BCG with i.n. ChAdOx1.PPE15 improved its protective efficacy, in agreement with previous published results (14). Vaccination with ChAdOx1.5Ag or ChAdOx1.5Ag-MVA.5Ag reduced the spleen bacterial load even further, but the two groups had comparable CFU.

## 4 Discussion

In this study, we vaccinated mice with an i.n. ChAdOx1 vector, utilizing its natural tropism for the lungs, in order to induce mucosal responses. Building upon previous evidence that ChAdOx1.PPE15, from the Esx-5a family of *M.tb*, was protective in mice, we investigated the efficacy of the remaining three members of this family (14). Initially, we administered i.n. ChAdOx1 vectors expressing each antigen and observed the induction of both mucosal and systemic immune responses, with one of the antigens, ChAdOx1.esxI, able to also significantly improve BCG efficacy. We subsequently combined the four Esx-5a antigens with the immunogenic Ag85A, into a single construct (5Ag), to test whether there was a synergistic protective effect. The 5Ag construct was inserted into ChAdOx1 and in MVA, a vector more likely to induce a strong CD4+ response (31). ChAdOx1.5Ag i.n. was tested alone (C) and in a heterologous boost combination with i.d. MVA.5Ag (C-M) for immunogenicity and protection. Previous data using Ag85A demonstrated that MVA was more protective when used to boost ChAdOx1, compared to when administered on its own (29). For this reason, MVA.5Ag was only tested as a booster vaccine in the present study. ChAdOx1.5Ag induced strong CD4+ and CD8+ T-cell antigen-specific responses in the lung, and although a further boost with MVA.5Ag did not further improve these responses in the lung, it did in the spleen. Both C and C-M, significantly improved the efficacy of BCG in both the lung and spleen; however, protection was equivalent to ChAdOx1.PPE15 in the lungs, with a trend for improved protection in the spleen.

The Esx-5a gene cluster was duplicated from Esx-5 three times, so one would expect a degree of similarity between the corresponding antigens in the different regions. The corresponding antigen of PPE15 in cluster Esx-5b is PPE18, one of the antigens in the promising M72/AS01e vaccine candidate currently undergoing clinical testing (8). PPE18 has been shown to have a role in *M.tb* virulence, as *M.tb* PPE18 knockout strain was less pathogenic in mice (32). Similar to PPE18, PPE15 has a highly conserved N-terminal domain, and a more variable C-terminal domain. Studies on the sequence variability of PPE18 revealed that the conserved N-terminal part might play a functional role, such as interaction with other proteins (33). In support of this, PPE15 has been shown to form a heterodimer with PE8 and, along with other members of the Esx-5a, mediate Esx-5 in secreting PE/PPE proteins (27, 34). The PE member of Esx-5c, PPE65, might therefore also be a protective antigen and a good vaccine candidate.

Unlike PE13 and PE32 of Esx-5b and Esx-5c, PE8 is much bigger in size (275 vs. 99 amino acids). Evidence suggests that the first 99 amino acids of PE8 are responsible for the interaction with PPE15, but the function of the rest of the sequence remains unknown (34). Sequence similarity search revealed that PE8 is the same size and has a high degree of homology to PE27, the largest PE member (excluding PE_PRGRS). PE27 has been shown to play an important role in *M.tb* virulence and to associate with PPE43, which itself has 69% homology to PPE15 (35). These data highlight the importance of these PE/PPE family of proteins in *M.tb* function and their ability to engage with the Th1 system, making them promising antigens for inclusion in vaccine candidates.

Esx antigens have been shown to be well recognized by human CD8+ T cells during TB infection, and by using delivery platforms that promote a CD8+ response, they might enhance this capacity (15). In support of this, esxI in ChAdOx1 was able to induce a very strong antigen-specific CD8+ T-cell response (**Figure 2**). There is more than 90% similarity between the esx genes members Esx-5 and 5a-c, which might explain their immunodominance and importance as vaccine candidates (36). The esx genes from the Esx-5c cluster, esxW and esxV, are present in the ID93/GLA-SE vaccine candidate, which previously showed good efficacy in animal models and is currently being evaluated in clinical trials (37, 38).

Inducing immune responses at the site of infection might be a more successful vaccination strategy, when it comes to respiratory pathogens such as SARS-Cov2 and TB (39). Recently, ChAdOx1 nCoV-19, expressing SARS-CoV-2 antigen, was administered i.n. in a phase I trial, and although it was safe and well tolerated, it induced weak immune responses (40). Alternative delivery devices could result in improved immune responses. *M.tb* is transmitted via aerosol and in droplet sizes lower than 5 µm, more likely to be deposited in the lower respiratory airways in humans (41, 42). Aerosolized delivery of vaccines has the potential to induce immune responses at the site of *M.tb* deposition and infection. ChAdOx1.85A was safely administered to humans via aerosol and was able to induce lung mucosal and systemic immune responses, highlighting the feasibility of this approach (Govender L. et al, NCT04121494).

Intranasal ChAdOx1.5Ag induced a strong mucosal response and is a good vaccine candidate to complement the weak mucosal immunogenicity of BCG (43, 44). Additionally, a further systemic boost with MVA.5Ag will improve the waning systemic BCG responses (45). Although the efficacy was comparable between BCG-ChAdOx1.5Ag and BCG-ChAdOx1.5Ag-MVA.5Ag, a longer interval between BCG and systemic MVA vaccination might have allowed a more sufficient boost to the waning efficacy of BCG (46).

Overall, these data justify the progression of these candidates to the next stage of vaccine development (47).

## Data availability statement

The datasets presented in this study can be found in online repositories. The names of the repository/repositories and accession number(s) can be found in the article/**Supplementary Material**.

## Ethics statement

The animal study was approved by Animal Welfare and Ethical Review Board (AWERB) University of Oxford. The study was conducted in accordance with the local legislation and institutional requirements.

## Author contributions

ES: Conceptualization, Data curation, Formal Analysis, Investigation, Methodology, Supervision, Writing – original draft, Writing – review & editing. NP: Investigation, Methodology, Writing – review & editing. OS: Investigation, Methodology, Writing – review & editing. AR: Investigation, Methodology, Writing – review & editing. MK: Investigation, Methodology, Writing – review & editing. HM: Conceptualization, Funding acquisition, Investigation, Resources, Supervision, Writing – review & editing.
