# Supplementary figures and images for: A five-antigen Esx-5a fusion delivered as a prime-boost regimen protects against M.tb challenge
Source: Front Immunol. 2023 Oct 5;14:1263457. doi: 10.3389/fimmu.2023.1263457 (PMC10585038; doi:10.3389/fimmu.2023.1263457)

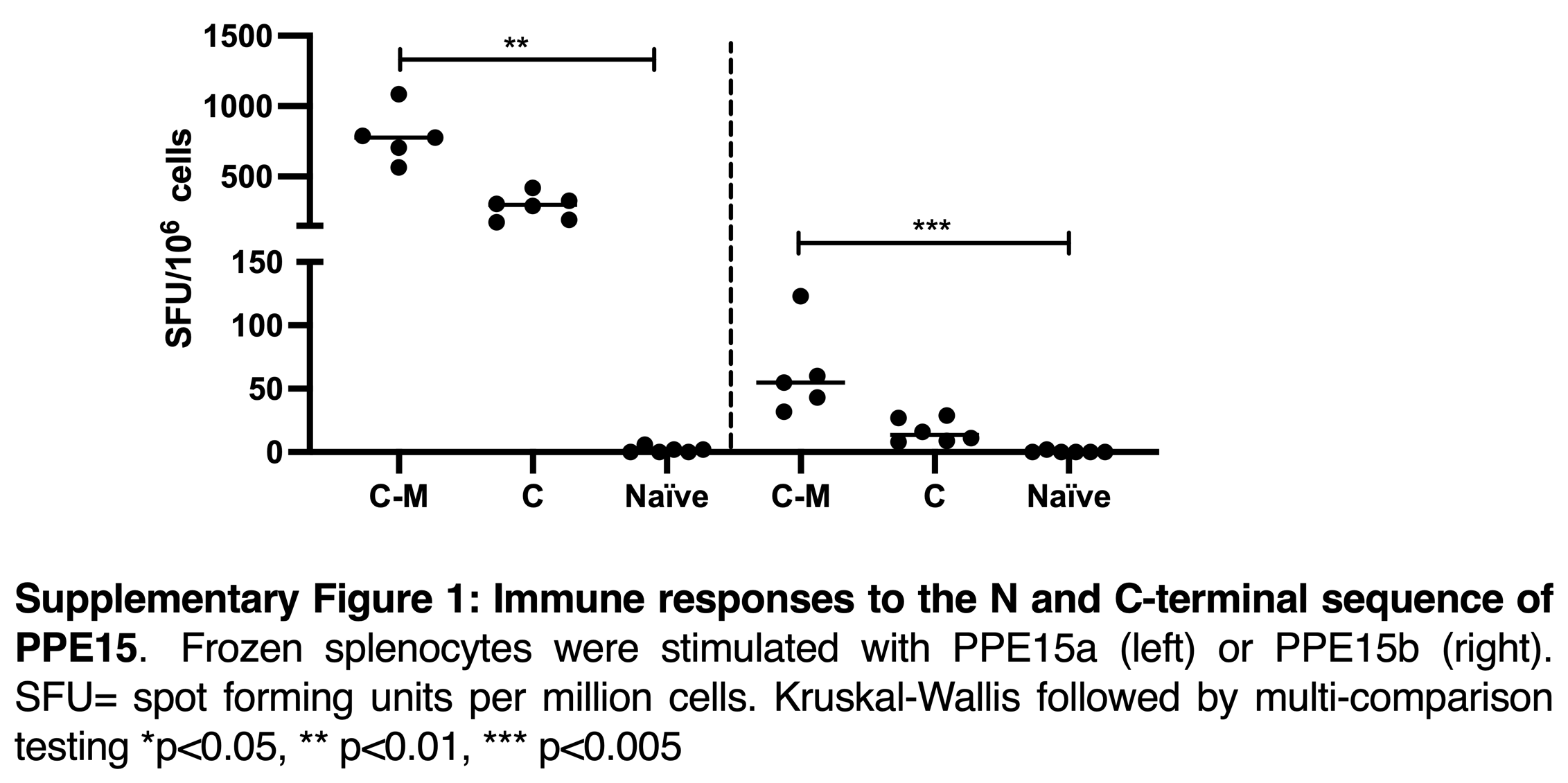

Supplement: Supplementary file 1 [file Image_1.tiff]

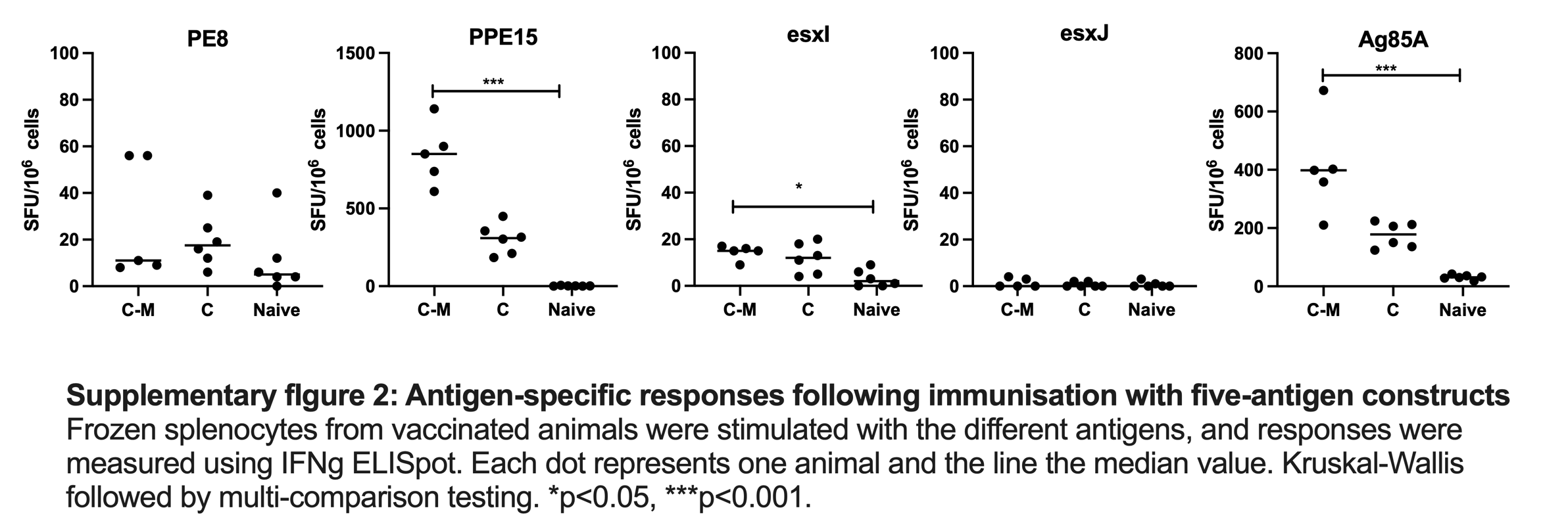

Supplement: Supplementary file 2 [file Image_2.tiff]
